# Circulating microRNA expression profile and systemic right ventricular function in adults after atrial switch operation for complete transposition of the great arteries

**DOI:** 10.1186/1471-2261-13-73

**Published:** 2013-09-16

**Authors:** Clare TM Lai, Enders KO Ng, Pak-cheong Chow, Ava Kwong, Yiu-fai Cheung

**Affiliations:** 1 Division of Paediatric Cardiology, Department of Paediatrics and Adolescent Medicine, The University of Hong Kong, Hongkong, China; 2 Department of Surgery, The University of Hong Kong, Hong Kong, China; 3 Division of Paediatric Cardiology, Department of Paediatrics and Adolescent Medicine, The University of Hong Kong, Queen Mary Hospital, 102, Pokfulam Road, Hong Kong, China

**Keywords:** MicroRNA, Transposition of the great arteries, Atrial switch, Systemic ventricular function

## Abstract

**Background:**

Data on the use of circulating microRNAs (miRNAs) as biomarkers of cardiovascular diseases are emerging. Little, however, is known on the expression profile of circulating of microRNAs in congenital heart malformations with a systemic right ventricle that is prone to functional impairment. We aimed to test the hypothesis that circulating miRNA profile is altered in patients late after atrial switch operation for complete transposition of the great arteries (TGA) and further explored possible relationships between alteration of circulating miRNAs and systemic ventricular contractility.

**Methods:**

Circulating miRNA expression profiling of serum samples from 5 patients and 5 healthy controls was performed. The results were validated in 26 patients and 20 controls using real-time quantitative reverse-transcription polymerase chain reaction for candidate miRNAs with fold changes >3 by expression profiling. Systemic ventricular myocardial acceleration during isovolumic contraction (IVA) was determined by colour tissue Doppler echocardiography.

**Results:**

Compared with controls, patients had significantly lower systemic ventricular IVA (p = 0.002). Of the 23 upregulated miRNAs identified by profiling, 11 were validated to be increased in patients compared with controls: miR-16, miR-106a, miR-144*, miR-18a, miR-25, miR-451, miR-486-3p, miR-486-5p, miR-505*, let-7e and miR-93. Among the validated 11 miRNAs, miR-18a (r = −0.45, p = 0.002) and miR-486-5p (r = −0.35, p = 0.018) correlated negatively with systemic ventricular IVA for the whole cohort.

**Conclusions:**

A distinct serum miRNA expression signature exists in adults with complete TGA after atrial switch operation, with serum miR-18a and miR-486-5p being associated with systemic ventricular contractility.

## Background

MicroRNAs (miRNAs) are small non-coding single-stranded RNAs with 19 to 24 nucleotides. These form complementary pair with specific target mRNAs to negatively regulate their expression via translational repression or degradation [1]. While alteration of tissue miRNA expression has been well documented in malignancies [2,3], the role of miRNA in cardiovascular development [4], cardiac remodeling and heart failure [5,6], and myocardial injury [7] is beginning to be recognized.

In determining the potential role of miRNAs in cardiovascular diseases, earlier studies have reported distinct profiles of differential tissue miRNA expression in human [8,9] and experimental models of heart failure [6,10]. Further cardiac tissue-based studies have provided evidence of involvement of selective miRNAs in myocardial hypertrophy [11,12], regulation of cardiac apoptosis [13], regulation of cytoskeleton of cardiomyocytes and cardiac extracellular matrix [11], and neurohormonal activation [14,15], all of which contribute to adverse cardiac remodeling.

Recent studies have shown that miRNAs released into the blood stream are stable and measurable [16,17]. Importantly, data in human subjects on the use of a signature circulating miRNAs as novel markers of cardiovascular diseases are emerging. Elevation of plasma cardiac-specific miRNA-208a has been shown to be potentially useful for early diagnosis of myocardial infarction [18], while plasma miR423-5p has also been found to be increased in heart failure patients with reduced left ventricular (LV) ejection fraction [19]. Additionally, a signature circulating miRNA expression profile has been reported in hypertensive patients [20]. These encouraging data, albeit limited to date, provide evidence that circulating miRNAs may be potential biomarkers of cardiovascular diseases, in particular LV diseases.

Little, however, is known on the expression profile of circulating microRNAs in congenital heart malformations with a systemic right ventricle. In patients with complete transposition of the great arteries (TGA) after atrial switch operation, late systemic right ventricular (RV) dysfunction is well documented [21,22]. There is further evidence to suggest that deterioration of systemic RV function and clinical status is progressive [22,23]. In this study, we tested the hypothesis that the circulating miRNA profile is altered in patients late after atrial switch operation and further explored the relationships between alteration of circulating miRNAs and systemic ventricular contractility.

## Methods

### Subjects

Twenty-six patients with complete TGA who had undergone Mustard or Senning procedure were recruited. The following demographic and clinical variables were collected: age at study, associated cardiac abnormalities, age at and type of operation, duration of follow-up since surgical repair, and current cardiac medications. Twenty healthy subjects were recruited as controls. The Institutional Review Board of The University of Hong Kong/Hospital Authority West Cluster, Hong Kong, approved the study and all study subjects gave written, informed consent.

### Serum collection and small RNA isolation

Blood obtained by venepuncture was centrifuged at 3000 rpm for 15 minutes immediately after collection. The centrifuged serum was transferred to another tube and re-spun once again to remove further debris. The serum samples were stored at −80°C until assay. Total RNA was extracted using Trizol LS reagent (Invitrogen, Carlsbad, California, USA) and miRNeasy Mini Kit (Qiagen, Hilden, California, USA) according to manufacturer’s in-structions with minor modifications [24]. Briefly, 500 μl of serum was mixed with 1 ml Trizol LS reagent and the aqueous phase of the mixture was transferred to a new tube and centrifuged after addition of chloroform. 1.5 volume of absolute ethanol was added to the aqueous phase and the mixture was applied into miRNeasy spin column as per manufacturer’s instructions. RNA was eluted with 30 μl RNase-free water. The concentration of RNA was quantified by NanoDrop ND-1000 Spectrophotometer (Nanodrop, Wilmington, Delaware, USA).

### MiRNA profiling

To study differential miRNA expression in TGA patients post atrial switch operation, we performed miRNA expression profiling of the serum samples from 5 patients and 5 age- and sex-matched controls using TaqMan Low Density Array Human MicroRNA Panel (Applied Biosystems, CA, USA), which contains >754 well-established miRNA assays including the respective reverse-transcription primers, PCR primers, and TaqMan probe. These 5 patients had the worst systemic RV function as assessed by echocardiography. For each array assay, 80 ng/μl serum miRNA was used for the reverse-transcription reaction with a pool of Megaplex RT primers. TaqMan-based qPCR profiling was performed using TaqMan Universal PCR Master Mix (Applied Biosystems) in ABI PRISM 7900 HT system (Applied Biosystems) according to the manufacturer’s instructions. U6 small nuclear RNA in the array was selected as the internal normalization transcript. The Δ cycle of threshold (Ct) was calculated by subtracting the averaged Ct values of U6 from the Ct values of the miRNA of interest. ΔΔCt was then calculated by subtracting ΔCt of the control from ΔCt of the sample. Fold change of the miRNA was calculated by the equation: 2^−ΔΔCt^. Up- and down-regulation of miRNAs with fold changes >3 were selected for validation in the entire patient cohort.

### Real-time quantitative reverse-transcription polymerase chain reaction

To validate the findings of miRNA profiling, we measured the expression levels of differentially expressed miRNAs using Real-Time Quantitative Reverse-Transcription Polymerase Chain Reaction (qRT-PCR) in all of the 26 patients and 20 controls. SYBR green qRT-PCR assay was used for individual miRNA quantification [24]. Briefly, 30 ng of serum RNA was polyadenylated by poly(A) polymerase and reversely transcribed using miScript Reverse Transcription Kit (Qiagen) according to manufacturer’s instructions. Real-time qPCR was performed using miScript SYBR Green PCR Kit (Qiagen) in ABI PRISM 7900 Real-time PCR system (Applied Biosystems). Amplification was performed with the miScript Universal primer provided by manufacturer and the miRNA-specific forward primers. The miRNA-specific primer sequences for qRT-PCR were listed in Additional file 1: Table S1. The amplification profile was 95°C for 15 minutes, followed by 45 cycles of 94°C for 15 seconds, 55°C for 30 seconds, and 70°C for 30 seconds. At the end of the PCR cycles, melting curve analyses were performed. The expression levels of miRNAs were normalised to U6 small nuclear RNA (RNU6B) level [24]. The fold change of the miRNA was calculated as aforementioned.

### Echocardiographic assessment

Transthoracic echocardiography was performed using the Vivid 7 ultrasound system (General Electric Vingmed Ultrasound, Horten, Norway). Echocardiographic recordings were made in three cardiac cycles and the average was used for statistical analysis. Offline analyses were performed using the EchoPAC software (General Electric, Horten, Norway). Colour tissue Doppler echocardiography was used to measure the relative load independent index of systolic function, myocardial acceleration during isovolumic contraction (IVA) [25], of the systemic ventricle. Colour-flow mapping was used for the assessment tricuspid regurgitation and baffle stenosis or leakage in patients.

### Statistical analysis

Data are presented as mean ± standard deviation or median (range) as specified. Demographic variables and echocardiographic parameters were compared using unpaired Student’s *t* test and Fisher’s exact test where appropriate. Serum miRNA expression levels between patients and controls were compared using Mann–Whitney test. Relationships between logarithmically transformed serum miRNA levels and systemic ventricular IVA were determined by Pearson correlation analysis. All p values are two-sided and a p value < 0.05 was considered statistically significant. All statistical analyses were performed using SPSS 16.0 (SPSS Inc., Chicago IL, USA).

## Results

### Subject characteristics

The demographic and clinical characteristics of all subjects are summarized in Table 1. Of the 26 patients, 22 had undergone Senning operation while 4 had Mustard procedure. Eight patients had closure of an associated ventricular septal defect. At the time of study, 5 patients were taking cardiac medications and were in New York Heart Association functional class II. The remaining 21 patients were in functional class I. There were no significant differences in age, sex distribution, and body size between patients and controls for both the cohort in the profiling study and the entire cohort included in the validation part (all p > 0.05).

**Table 1:** Demographic and clinical characteristics of patients and controls

	***Microarray***			***qRT-PCR validation***		
	**Patients (n = 5)**	**Controls (n = 5)**	***p***	**Patients (n = 26)**	**Controls (n = 20)**	***p***
Age at study (years)	26.5 ± 5.1	24.5 ± 6.3	0.60	25.3 ± 3.2	25.0 ± 4.3	0.82
Gender (M:F)	3:2	3:2	1.0	17:9	11:9	0.82
BMI (kg/m^2^)	19.9 ± 1.5	23.3 ± 3.9	0.12	21.8 ± 3.3	21.6 ± 3.3	0.84
BSA (m^2^)	1.66 ± 0.09	1.77 ± 0.22	0.30	1.67 ± 0.14	1.70 ± 0.21	0.57
Age at surgery (years)	2.24 ± 2.26	-		1.90 ± 2.26	-	
Type of operation (Mustard: Senning)	1:4	-		4:22	-	
Closure of VSD	2	-		8	-	
Medical treatment (n)	(2)	-		(5)	-	
- ACEI	2	-		4	-	
- ARB	2	-		3	-	
- furosemide	1	-		1	-	
- spironolactone	1	-		1	-	
- aspirin	1	-		1	-	
- digoxin	0	-		1	-	

*ACEI:* = angiotensin-converting enzyme inhibitors, *ARB:* = angiotensin II receptor blockers, *BMI:* = body mass index, *BSA:* = body surface area, *qRT-PCR:* = real-time quantitative reverse-transcription polymerase chain reaction, *VSD:* = ventricular septal defect.

### Cardiac status in patients

Tricuspid regurgitation was severe in 1 patient, trivial to mild in 19, and absent in 6. Mild baffle leak was found in 1 patient, while mild subpulmonary stenosis was noted in 2. History of cardiac arrhythmias was documented in 4 patients, with 1 each having sinus node dysfunction, first degree heart block, atrial flutter, and infrequent ventricular ectopics. All patients were in sinus rhythm at the time of study. Compared with controls, patients had significantly reduced systemic ventricular IVA (1.06 ± 0.42 m/s^2^ vs 1.45 ± 0.39 m/s^2^, p = 0.002).

### Identification of differentially expressed serum miRNAs in patients

**Figure 1:**
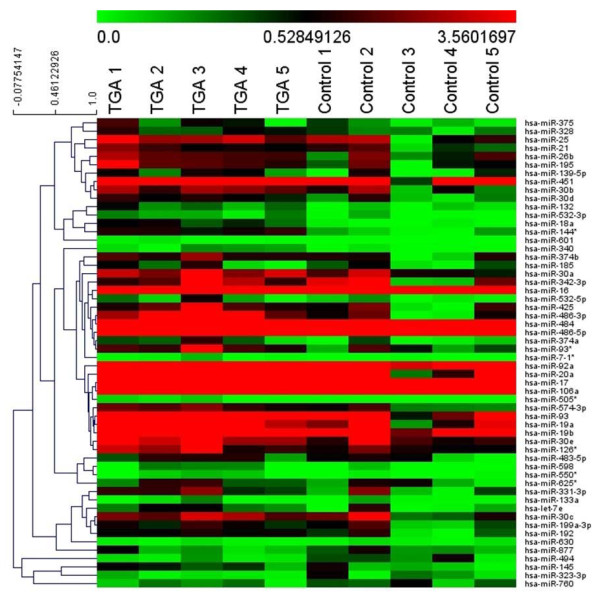
**Profiling of serum microRNAs in 5 patients after atrial switch operation for transposition of the great arteries (TGA) and 5 healthy control subjects.** miRNAs with fold changes >2 are shown in the heat map. The expression of miRNA is hierarchically clustered on the y-axis. Colour scale is shown on the top with green indicating downregulation and red upregulation.

Serum miRNA expression profiling was performed using serum samples from 5 patients and 5 controls matched for age and sex (Table 1). The level of RNU6B used for normalization was similar between patients and controls (32.3 ± 0.4 vs 32.4 ± 0.5, p = 0.54). The levels of serum miRNAs differed between patients and controls as illustrated in the heat map (Figure 1). One hundred miRNAs with fold change >1 and RNU6B levels are shown in Additional file 2: Table S2. Using a 3-fold expression difference as a cut-off level, 23 miRNAs were found to be significantly up-regulated and 1 miRNA significantly down-regulated in patients compared with controls (Table 2). As miR-505* was expressed only in patients, the absolute fold-change could not be calculated.

**Table 2:** Up- and down-regulated miRNAs as defined by fold changes >3 by expression profiling in patients compared with control subjects

**MicroRNA**	**MirBase no**	**Average fold change**
miR-505*	MIMAT0004776	↑ in patients only
miR-144*	MIMAT0004600	15.08
miR-18a	MIMAT0000072	14.9
miR-486-3p	MIMAT0004762	8.67
miR-20a	MIMAT0000075	7.53
miR-451	MIMAT0001631	6.04
miR-486-5p	MIMAT0002177	5.45
miR-374a	MIMAT0000727	5.20
miR-16	MIMAT0000069	5.09
miR-375	MIMAT0000728	4.80
miR-331-3p	MIMAT0000760	4.50
Hsa-let-7e	MIMAT0000066	4.47
miR-25	MIMAT0000081	4.01
miR-93*	MIMAT0004509	3.76
miR-93	MIMAT0000093	3.62
miR-92a	MIMAT0000092	3.58
miR-30b	MIMAT0000420	3.56
miR-19a	MIMAT0000073	3.53
miR-17-5p	MIMAT0000070	3.39
miR-106a	MIMAT0000103	3.28
miR-30d	MIMAT0000245	3.22
miR-574-3p	MIMAT0004795	3.18
miR-19b	MIMAT0000074	3.08
miR-494	MIMAT0002816	0.27

* is the notation for some of the miRs.

↑ in patients only’ literally ‘increased in patients only.

### Larger sample size validation

**Figure 2:**
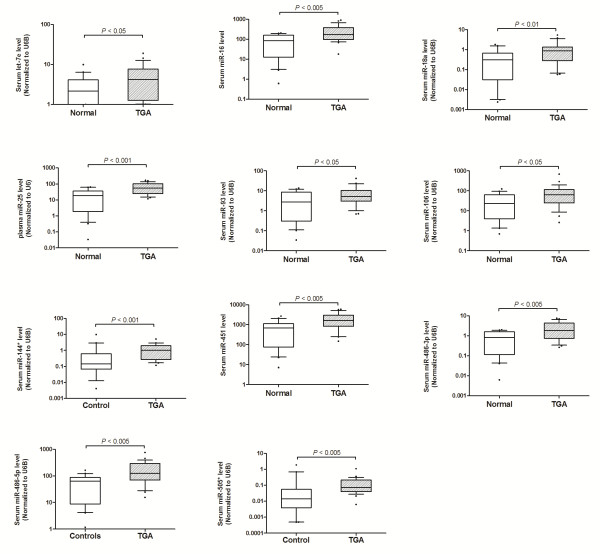
**Validation of serum microRNAs levels in patients and controls.** Levels of miRNA expression are normalized to RNU6B level. Horizontal line represents the median.

To validate the profiling results, qRT-PCR was performed to measure the serum expression levels of the 24 miRNAs in 26 patients and 20 controls. For the whole cohort, there was similarly no significant difference in RNU6B level between patients and controls (28.7 ± 1.3 vs 28.8 ± 1.6, p = 0.79). Among the 23 up-regulated miRNAs, 11 were significantly elevated in patients compared with controls: miR-16, miR-106a, miR-144*, miR-18a, miR-25, miR-451, miR-486-3p, miR-486-5p, miR-505*, let-7e and miR-93 (Figure 2). However, down-regulation of miR-494 was not confirmed by qRT-PCR.

### Factors associated with up-regulated serum miRNAs

**Figure 3:**
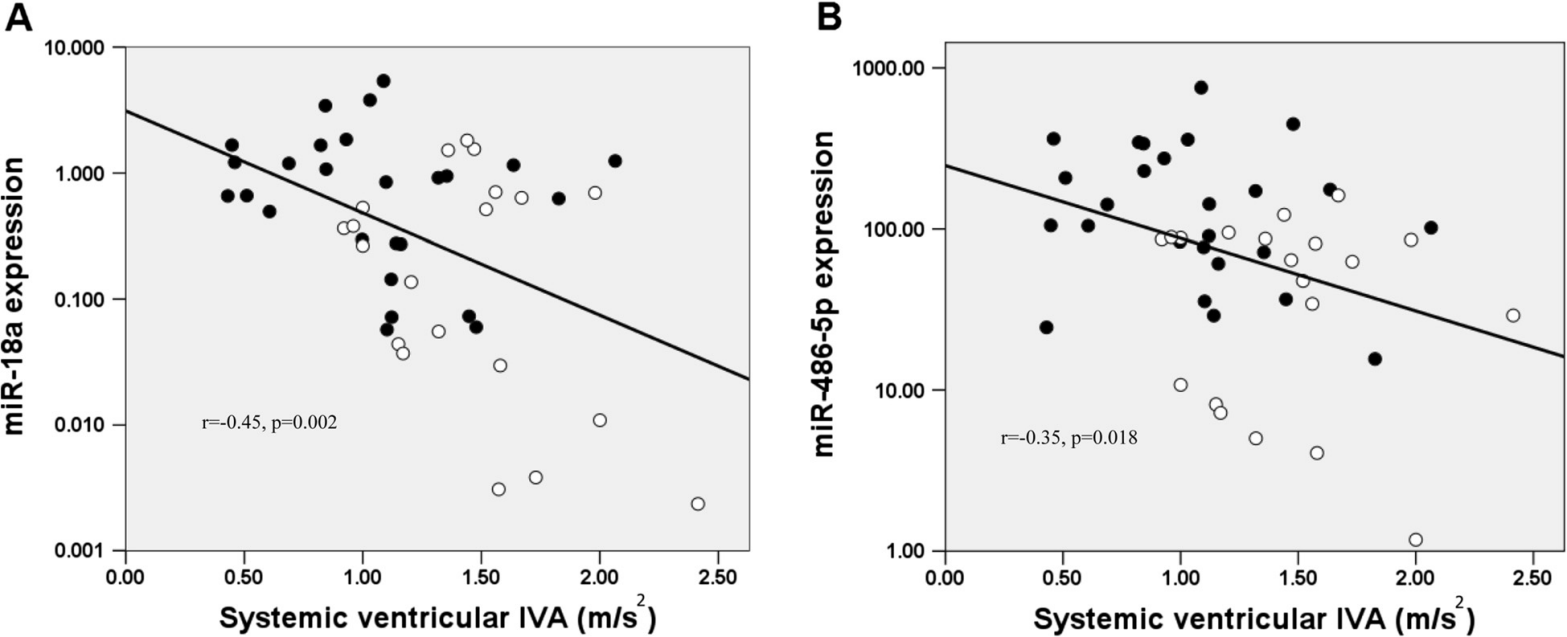
**Scatter plots showing negative correlation between systemic ventricular myocardial acceleration during isovolumic contraction (IVA) and (A) serum miR-18a and (B) miR-486-5p expressions.** Closed and open dots represent patients and controls, respectively.

For the whole cohort, IVA correlated negatively with serum miR-18a (r = −0.45, p = 0.002) and miR-486-5p levels (r = −0.35, p = 0.018) (Figure 3). Furthermore, serum levels of miR-18a and miR-486-5p were positively correlated (r = 0.575, p < 0.001) (Additional file 3: Figure S1). None of validated miRNAs were associated with baseline demographic characteristics except for miR-144*, the level of which correlated with body mass index (r = 0.32, p = 0.03) (Additional file 4: Table S3).

## Discussion

The present study demonstrates a distinct serum miRNA expression signature in adult patients with complete TGA after atrial switch operation. Expression profiling of circulating miRNA revealed significant upregulation of 23 miRNAs and downregulation of 1 miRNA, while only 11 of the upregulated miRNAs were validated in a larger patient cohort using qRT-PCR. Importantly, significant negative correlations were identified between systemic ventricular IVA, a relative load-independent index of systemic ventricular contractility, and two miRNAs miR-18a and miR-486-5p.

There is paucity of data on the expression profile of circulating microRNAs in congenital heart malformations with a systemic right ventricle. In the only study published to date, Tutarel et al. showed no difference in the levels of circulating miR-423-5p between adults after atrial repair of TGA and healthy subjects [26]. To our knowledge, this is the first study using expression profiling followed by validation of differentially expressed serum miRNAs to explore potential alteration of circulating miRNA profile in patients late after atrial switch operation.

Of the 11 validated miRNAs, several had been reported to associate with cardiovascular and cerebrovascular diseases. Up-regulation of peripheral blood let-7e has been shown in large and small artery stroke, cardioembolic stroke and stroke with undetermined causes, while expression of miR-16, miR-106a, miR-25 and miR-93 were found to be differentially increased in patients with small artery stroke only [27]. In hypertensive patients, plasma expression of let-7e has been demonstrated to be elevated in profiling studies [20]. In a rodent model of pulmonary hypertension, miR-451 in lung tissues showed a strong upregulation in hypoxic samples [28], while in children with aortic stenosis undergoing aortic valve replacement, differential upregulation of ventricular let-7e and miR-93 has been reported [8].

The differentially expressed circulating miRNAs identified in the present study appear to be involved in biologic processes that are of relevance to our patients: angiogenesis (miR-16 and miR-18a) [29,30], hypoxia regulation (let-7e, miR-16, miR106a and miR-93) [31], and apoptosis and cell cycle control (miR-16) [32,33]. In this regard, it is worthwhile noting that abnormal myocardial perfusion and reduced coronary flow reserve [34,35] are well documented in patients after atrial switch operation. It would also be interesting to determine the level of vascular endothelial growth factors in patients in future studies. Our recent finding of increased circulating annexin A5 lends support to possible increased apoptotic process in these patients [36] Gene prediction programmes [37] identify biological pathways of cardiac significance, which include MAPK signaling [38], Wnt signaling [39], and TGF beta signaling [40].

Progressive systemic RV dysfunction in patients after Senning and Mustard operation remains an important issue of concern [21,22]. MicroRNAs may play a role in LV remodeling process through regulating the gene networks that modulate cardiomyocyte apoptosis and myocardial fibrosis [41]. While the precise mechanism of systemic RV dysfunction remains elusive, our novel findings of negative relationships between expression of serum miR-18a and miR-486-5p and systemic ventricular IVA suggests a possible role of dysregulated microRNAs.

The contractile apparatus of the systemic right ventricle may potentially be jeopardized by ischaemia [34,35] and apoptosis [36]. miR-18a has been shown to downregulate Ataxia Telangiectasia Mutated (ATM) expression and reduce DNA damage repair ability [42]. Further studies have unveiled a protective role ATM in cardiac apoptosis [43]. There are also suggestions that our other validated candidate miR-486-5p, which is enriched in cardiac and skeletal muscles, may have a role in modulating cardiac contractile function [44]. Overexpression of miR-486 has been reported to enhance muscle phosphoinositide-3-kinse (PI3K)/Akt signaling by reducing the upstream negative regulator, phosphatase and tensin homolog (PTEN) [44]. Of relevance is the demonstration of decreased cardiac contractility with pharmacological inhibition of PTEN and in cardiac muscle specific PTEN knockout mice [45,46]. Furthermore, chronic overexpression of Akt has been shown to cause maladaptive hypertrophy of the left ventricle with dilation and contractile dysfunction [47]. Although we have not determined the levels of brain natriuretic peptide, a biomarker of ventricular dysfunction, in the present study, we [48] and others [49] have reported previously the elevation of brain natriuretic peptide in patients after atrial repair with impaired systemic RV function.

With regard to the cardiac extracellular matrix, the cardiomyocyte-derived miR-18a has been reported to play a role in age-related heart failure through modulation of matrix protein levels [50]. Transfection of miR-25, another of our validated miRNA candidate, in cardiac fibroblast has also been demonstrated to decrease collagen gene expression [51]. Indeed, our recent findings of increased tumour necrosis factor and absence of increase in circulating biomarkers of collagen biosynthesis in patients with TGA after atrial switch operation implicate reduced myocardial collagen synthesis [36]. Hence, reduction of matrix tensile strength may be a cause of progressive RV dilation in post Senning and Mustard patients.

Taken together, our novel finding of a distinct circulating miRNA profile sheds light on the pathogenesis of systemic RV dysfunction in patients after atrial switch operation from an entirely new perspective. There is no doubt, however, that the findings are preliminary and that the molecular mechanisms of upregulation of circulating miRNAs in our patients require further investigations. In this regard, inclusion of additional patient cohorts with subpulmonary right ventricles operating under relatively normal pressure, as in TGA patients after arterial switch operation, and increased pressure, as those with severe pulmonary stenosis, may shed more light on the underlying mechanism. Another important limitation of the present study is that we cannot ascertain the source in our patients of the upregulated circulating miRNAs, which may occur as a result of cellular damage or secretion [52]. Nonetheless, De Rosa et al. have recently provided convincing evidence of the cardiac source of circulating miRNAs associated with acute coronary syndromes [53]. Furthermore, despite the undetermined source, circulating miRNAs may function as potential paracrine mediator of cardiovascular disease [54].

## Conclusion

In conclusion, the present study provides the first evidence of an altered circulating miRNA expression profile in adult congenital heart patients with a systemic right ventricle after atrial switch operation. While we have validated the expression profiling results independently in a bigger cohort of subjects, these preliminary data need to be confirmed in a larger clinical population. Given the demonstrable relationships between serum miR-18a and miR-486-5p expressions and systemic ventricular contractility, further studies are warranted to explore the potential usefulness of circulating miRNAs as biomarkers for development of systemic RV dysfunction.

## Competing interests

The author(s) declare that they have no competing interests.

## Authors’ contributions

YFC has been involved in design of the study. CTML and PCC recruited the subjects. CTML and PCC have been involved in echocardiographic data acquisition. CTML carried out the experiments. EKON provided laboratory technical help. CTML processed the data. CTML performed the statistical analysis. YFC and CTML analysed and interpreted the data. YFC and CTML drafted and edited the manuscript. YFC, CTML, EKON and AK critically revised the manuscript. YFC supervised the study. All authors have read and agree to the manuscript as written.

## Pre-publication history

The pre-publication history for this paper can be accessed here:

http://www.biomedcentral.com/1471-2261/13/73/prepub

## Supplementary Material

Additional file 1: Table S1 MiRNA-specific primer sequences for qRT-PCR. Click here for file

Additional file 2: Table S2 MiRNAs with fold change > 1 and levels of RNU6B. Click here for file

Additional file 3: Figure S1 Scatter plot showing a positive correlation between serum levels of miR-18a and miR-486-5p. Click here for file

Additional file 4: Table S3 Correlations between miRNAs and demographic characteristics and systemic ventricular isovolumic acceleration. Click here for file
