# Supplementary material for: Circulating microRNA expression profile and systemic right ventricular function in adults after atrial switch operation for complete transposition of the great arteries
Source: BMC Cardiovasc Disord. 2013 Sep 16;13:73. doi: 10.1186/1471-2261-13-73 (PMC3847493; doi:10.1186/1471-2261-13-73)
Supplement: Additional file 1: Table S1 — MiRNA-specific primer sequences for qRT-PCR. [file 1471-2261-13-73-S1.pdf]

Supplementary Table 1 MiRNA-specific primer sequences for qRT-PCR

MicroRNAs	MirBase no.	Primer sequences (5' to 3')
miR-505*	MIMAT0004776	GGAGCCAGGAAGTATTGATGT
miR-144*	MIMAT0004600	GGATATCATCATATACTGTAAG
miR-18a	MIMAT0000072	TAAGGTGCATCTAGTGCAGATA
miR-486-3p	MIMAT0004762	GGGGCAGCTCAGTACAGGA
miR-20a	MIMAT0000075	TAAAGTGCTTATAGTGCAGGTA
miR-451	MIMAT0001631	AAACCGTTACCATTACTGAGTT
miR-486-5p	MIMAT0002177	CTGTACTGAGCTGCCCCGA
miR-374a	MIMAT0000727	TTATAATACAACCTGATAAGTG
miR-16	MIMAT0000069	TAGCAGCACGTAAATATTGGCG
miR-375	MIMAT0000728	TTGTTCGTTCGGCTCGCGTG
miR-331-3p	MIMAT0000760	CCCCTGGGCCTATCCTAGA
Hsa-let-7e	MIMAT0000066	TGAGGTAGGAGGTTGTATAGTT
miR-25	MIMAT0000081	ATTGCACTTGTCTCGGTCTG
miR-93*	MIMAT0004509	CTGCTGAGCTAGCACTTCCC
miR-93	MIMAT0000093	AAAGTGCTGTTCGTGCAGGTA
miR-92a	MIMAT0000092	TATTGCACTTGTCCCGGCCTG
miR-30b	MIMAT0000420	TGTAAACATCCTACACTCAGCT
miR-19a	MIMAT0000073	TGTGCAAATCTATGCAAAACTGA
miR-17-5p	MIMAT0000070	CAAAGTGCTTACAGTGCAGGTA
miR-106a	MIMAT0000103	AAAGTGCTTACAGTGCAGGTA
miR-30d	MIMAT0000245	TGTAAACATCCCCGACTGGAA
miR-574-3p	MIMAT0004795	ACGCTCATGCACACACCCAC
miR-19b	MIMAT0000074	TGTGCAAATCCATGCAAAACTGA
miR-494	MIMAT0002816	TGAAACATACACGGGAAACCTC
