# Supplementary material for: Circulating microRNA expression profile and systemic right ventricular function in adults after atrial switch operation for complete transposition of the great arteries
Source: BMC Cardiovasc Disord. 2013 Sep 16;13:73. doi: 10.1186/1471-2261-13-73 (PMC3847493; doi:10.1186/1471-2261-13-73)
Supplement: Additional file 2: Table S2 — MiRNAs with fold change > 1 and levels of RNU6B. [file 1471-2261-13-73-S2.pdf]

MicroRNA	MirBase #	Serum from 5 patients & 5 age-matched controls				Fold change
		Patients		Controls		
		Average Ct values	Normalized to U6	Average Ct values	Normalized to U6	
hsa-miR-18a	MIMAT0000072	33.78	0.4464	37.88	0.0299	14.93
hsa-miR-486-3p	MIMAT0004762	30.77	3.5772	34.09	0.4124	8.67
hsa-miR-20a	MIMAT0000075	28.39	18.6788	31.50	2.4818	7.53
hsa-miR-451	MIMAT0001631	27.82	27.7951	30.61	4.5991	6.04
hsa-miR-486-5p	MIMAT0002177	25.29	159.8507	27.94	29.3144	5.45
hsa-miR-374a	MIMAT0000727	34.27	0.3177	36.84	0.0611	5.20
hsa-miR-16	MIMAT0000069	24.56	264.5274	27.11	51.9953	5.09
hsa-miR-375	MIMAT0000728	33.43	0.5670	35.89	0.1183	4.80
hsa-miR-331-3p	MIMAT0000760	32.80	0.8762	35.17	0.1947	4.50
hsa-let-7e	MIMAT0000066	34.11	0.3549	36.47	0.0795	4.47
hsa-miR-25	MIMAT0000081	31.26	2.5591	33.46	0.6384	4.01
hsa-miR-93	MIMAT0000093	28.93	12.7968	30.99	3.5380	3.62
hsa-miR-92a	MIMAT0000092	28.08	23.1249	30.12	6.4564	3.58
hsa-miR-30b	MIMAT0000420	31.66	1.9394	33.69	0.5441	3.56
hsa-miR-19a	MIMAT0000073	30.24	5.1816	32.26	1.4673	3.53
hsa-miR-17	MIMAT0000070	27.07	46.6521	29.03	13.7633	3.39
hsa-miR-106a	MIMAT0000103	27.14	44.4266	29.05	13.5315	3.28
hsa-miR-574-3p	MIMAT0004795	32.13	1.4001	33.99	0.4409	3.18
hsa-miR-19b	MIMAT0000074	27.15	43.9781	28.98	14.2783	3.08
hsa-miR-483-5p	MIMAT0004761	33.39	0.5820	35.16	0.1958	2.97
hsa-miR-132	MIMAT0000426	34.44	0.2811	36.20	0.0953	2.95
hsa-miR-532-3p	MIMAT0002888	35.43	0.1418	37.17	0.0488	2.90
hsa-miR-145	MIMAT0000437	34.10	0.3553	35.77	0.1283	2.77
hsa-miR-342-3p	MIMAT0000753	31.48	2.1909	33.15	0.7933	2.76
hsa-miR-26b	MIMAT0000083	31.91	1.6228	33.57	0.5909	2.75
hsa-miR-484	MIMAT0002174	27.40	36.9500	29.06	13.4821	2.74
hsa-miR-425	MIMAT0003393	32.04	1.4896	33.67	0.5504	2.71
hsa-miR-340	MIMAT0004692	35.25	0.1610	36.88	0.0595	2.70
hsa-miR-328	MIMAT0000752	33.62	0.4971	35.24	0.1864	2.67
hsa-miR-195	MIMAT0000461	31.88	1.6663	33.47	0.6355	2.62
hsa-miR-374b	MIMAT0004955	32.30	1.2389	33.77	0.5135	2.41
hsa-miR-185	MIMAT0000455	34.23	0.3250	35.63	0.1415	2.30
hsa-miR-30c	MIMAT0000244	31.58	2.0497	32.96	0.9008	2.28
hsa-miR-133a	MIMAT0000427	36.58	0.0639	37.86	0.0302	2.12
hsa-miR-21	MIMAT0000076	32.73	0.9211	33.99	0.4414	2.09
hsa-miR-199a-3p	MIMAT0000232	33.24	0.6472	34.49	0.3116	2.08
hsa-miR-532-5p	MIMAT0002888	35.49	0.1356	36.75	0.0654	2.07
hsa-miR-139-5p	MIMAT0000250	33.84	0.4280	35.08	0.2076	2.06
hsa-miR-192	MIMAT0000222	33.30	0.6196	34.55	0.3005	2.06
hsa-miR-598	MIMAT0003266	34.58	0.2562	35.78	0.1277	2.01
hsa-miR-150	MIMAT0000451	30.02	6.0231	31.20	3.0603	1.97
hsa-miR-193b	MIMAT0002819	35.19	0.1671	36.29	0.0898	1.86
hsa-let-7g	MIMAT0000414	34.41	0.2870	35.50	0.1549	1.85
hsa-miR-20b	MIMAT0001413	31.16	2.7299	32.24	1.4836	1.84
hsa-miR-146a	MIMAT0000449	29.59	8.1321	30.67	4.4255	1.84
hsa-miR-191	MIMAT0000440	28.55	16.6436	29.61	9.2101	1.81
hsa-miR-345	MIMAT0000772	33.03	0.7476	34.08	0.4165	1.80
hsa-miR-26a	MIMAT0000082	32.62	0.9955	33.63	0.5686	1.75
hsa-miR-122	MIMAT0000421	31.26	2.5466	32.27	1.4547	1.75
hsa-miR-223	MIMAT0000280	23.84	437.8200	24.81	256.9354	1.70
hsa-miR-222	MIMAT0000279	28.99	12.2747	29.95	7.2927	1.68
hsa-miR-301a	MIMAT0000688	34.34	0.3027	35.25	0.1851	1.64
hsa-miR-106b	MIMAT0000680	32.49	1.0882	33.39	0.6699	1.62
hsa-miR-27a	MIMAT0000084	34.76	0.2255	35.63	0.1415	1.59
hsa-miR-15b	MIMAT0000417	35.09	0.1790	35.95	0.1136	1.58
hsa-miR-24	MIMAT0000080	29.52	8.5403	30.36	5.4839	1.56
hsa-miR-186	MIMAT0000456	31.38	2.3447	32.21	1.5143	1.55
hsa-miR-140-3p	MIMAT0004597	33.93	0.3998	34.74	0.2632	1.52
hsa-miR-422a	MIMAT0001339	36.75	0.0568	37.52	0.0382	1.49
hsa-miR-140-5p	MIMAT0000431	32.75	0.9106	33.51	0.6161	1.48
hsa-miR-320	MIMAT0000510	28.61	16.0711	29.34	11.0831	1.45
hsa-miR-126	MIMAT0000445	28.46	17.7309	29.19	12.3030	1.44
hsa-miR-125a-5p	MIMAT0000443	34.75	0.2278	35.42	0.1644	1.39
hsa-miR-410	MIMAT0002171	36.47	0.0690	37.13	0.0500	1.38
hsa-miR-590-5p	MIMAT0003258	34.89	0.2061	35.54	0.1505	1.37
hsa-miR-335	MIMAT0000765	36.96	0.0492	37.61	0.0360	1.37
hsa-miR-142-3p	MIMAT0000434	33.49	0.5422	34.12	0.4030	1.35
hsa-miR-29c	MIMAT0000681	36.21	0.0828	36.80	0.0631	1.31
hsa-miR-193a-5p	MIMAT0004614	35.93	0.1001	36.50	0.0775	1.29
hsa-miR-130a	MIMAT0000425	34.76	0.2249	35.26	0.1832	1.23
hsa-miR-125b	MIMAT0000423	34.78	0.2228	35.22	0.1888	1.18
hsa-miR-134	MIMAT0000447	36.75	0.0569	37.14	0.0497	1.15
hsa-miR-744	MIMAT0004945	34.70	0.2346	35.09	0.2061	1.14
hsa-miR-885-5p	MIMAT0004947	32.91	0.8121	33.24	0.7442	1.09
hsa-let-7b	MIMAT0000063	31.67	1.9267	31.97	1.7962	1.07
hsa-miR-652	MIMAT0003322	37.24	0.0405	37.53	0.0379	1.07
hsa-miR-454	MIMAT0003885	33.51	0.5375	33.73	0.5287	1.02
hsa-miR-197	MIMAT0022691	34.73	0.2304	34.60	0.2896	0.80
hsa-miR-28-3p	MIMAT0004502	34.08	0.3619	33.33	0.6993	0.52
hsa-miR-323-3p	MIMAT0000755	36.94	0.0499	35.78	0.1281	0.39
hsa-miR-494	MIMAT0002816	36.52	0.0666	34.85	0.2438	0.27
<u>For Plate A</u>						
MammU6-4395470		32.53		32.89		
MammU6-4395470		32.69		32.70		
MammU6-4395470		32.46		32.84		
MammU6-4395470		32.76		32.82		
Average		**32.61**		**32.81**		
hsa-miR-505*	MIMAT0004776	35.68	0.0753	Undetermined	Undetermined	upregulate in patients only
hsa-miR-144*	MIMAT0004600	32.25	0.8072	36.23	0.0535	15.08
hsa-miR-93*	MIMAT0004509	31.35	1.5148	33.32	0.4028	3.76
hsa-miR-550*	MIMAT0004800	36.30	0.0488	38.21	0.0136	3.59
hsa-miR-30d-4373059	MIMAT0000245	32.48	0.6886	34.23	0.2138	3.22
hsa-miR-30a	MIMAT0000087	30.40	2.9153	31.87	1.0989	2.65
hsa-miR-30e	MIMAT0000066	30.09	3.6302	31.53	1.3954	2.60
hsa-miR-601	MIMAT0003269	37.84	0.0168	39.28	0.0065	2.59
hsa-miR-7-1*	MIMAT0004553	36.99	0.0303	38.35	0.0123	2.46
hsa-miR-126*	MIMAT0000444	31.00	1.9218	32.27	0.8356	2.30
hsa-miR-625*	MIMAT0004808	32.90	0.5169	34.13	0.2294	2.25
hsa-miR-425*	MIMAT0001343	34.54	0.1651	35.21	0.1083	1.52
hsa-miR-151-3p	MIMAT0000757	31.39	1.4688	32.03	0.9823	1.50
hsa-miR-378	MIMAT0000732	31.52	1.3417	31.99	1.0127	1.32
hsa-miR-188-5p	MIMAT0000457	32.32	0.7722	32.61	0.6581	1.17
hsa-miR-630	MIMAT0003299	37.18	0.0266	36.95	0.0326	0.82
hsa-miR-887	MIMAT0004951	34.52	0.1680	33.81	0.2865	0.59
hsa-miR-760	MIMAT0004957	34.87	0.1321	33.81	0.2868	0.46
<u>For Plate B</u>						
MammU6-4395470		31.79			31.7182	
MammU6-4395470		32.03			32.2360	
MammU6-4395470		32.16			32.0882	
MammU6-4395470		31.81			31.9893	
Average		**31.95**			**32.0079**	
