# Supplementary figures and images for: Circulating microRNA expression profile and systemic right ventricular function in adults after atrial switch operation for complete transposition of the great arteries
Source: BMC Cardiovasc Disord. 2013 Sep 16;13:73. doi: 10.1186/1471-2261-13-73 (PMC3847493; doi:10.1186/1471-2261-13-73)

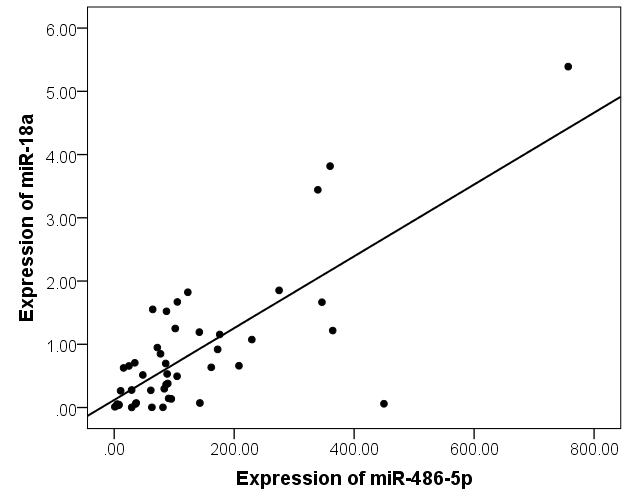

Supplement: Additional file 3: Figure S1 — Scatter plot showing a positive correlation between serum levels of miR-18a and miR-486-5p. [file 1471-2261-13-73-S3.tiff]
