# Supplementary material for: Circulating microRNA expression profile and systemic right ventricular function in adults after atrial switch operation for complete transposition of the great arteries
Source: BMC Cardiovasc Disord. 2013 Sep 16;13:73. doi: 10.1186/1471-2261-13-73 (PMC3847493; doi:10.1186/1471-2261-13-73)
Supplement: Additional file 4: Table S3 — Correlations between miRNAs and demographic characteristics and systemic ventricular isovolumic acceleration. [file 1471-2261-13-73-S4.pdf]

	miR-16	miR-106a	miR-144*	miR-18a	miR-25	miR-451	miR-486-3p	miR-486-5p	miR-505*	hsa-let-7e	miR-93
**Age**	r=0.116<br>p=0.443	r=0.009<br>p=0.954	r=0.121<br>p=0.422	r=-0.090<br>p=0.551	r=0.140<br>p=0.352	r=0.057<br>p=0.707	r=0.134<br>p=0.374	r=0.095<br>p=0.529	r=0.207<br>p=0.177	r=-0.024<br>p=0.874	r=0.111<br>p=0.463
**Gender**	r=0.114<br>p=0.449	r=0.167<br>p=0.268	r=0.045<br>p=0.767	r=0.049<br>p=0.747	r=0.292<br>p=0.049*	r=0.215<br>p=0.151	r=0.233<br>p=0.119	r=0.224<br>p=0.134	r=0.143<br>p=0.354	r=-0.057<br>p=0.708	r=0.205<br>p=0.171
**BMI**	r=0.209<br>p=0.163	r=0.049<br>p=0.744	r=0.321<br>p=0.030*	r=-0.177<br>p=0.239	r=0.157<br>p=0.297	r=0.166<br>p=0.271	r=0.069<br>p=0.649	r=0.066<br>p=0.663	r=0.205<br>p=0.181	r=-0.171<br>p=0.255	r=0.207<br>p=0.168
**Systemic ventricular IVA**	r=-0.182<br>p=0.226	r=-0.290<br>p=0.051	r=-0.022<br>p=0.886	r=-0.446<br>p=0.002*	r=-0.198<br>p=0.186	r=-0.176<br>p=0.241	r=-0.245<br>p=0.101	r=-0.348<br>p=0.018*	r=-0.039<br>p=0.802	r=-0.003<br>p=0.986	r=-0.156<br>p=0.299

BMI indicates body mass index; and IVA, isovolumetric acceleration.
